# Pacemaker Versus Defibrillator Therapy in Patients Eligible for Cardiac Resynchronisation Therapy: Evidence from the German Device Registry

**DOI:** 10.3390/jcm14041137

**Published:** 2025-02-10

**Authors:** Philipp S. Lange, Gerrit Frommeyer, Thomas Kleemann, Johannes Brachmann, Patrick Lugenbiel, Sebastian Reif, Matthias Hochadel, Jochen Senges, Lars Eckardt

**Affiliations:** 1 Division of Electrophysiology, Department of Cardiovascular Medicine, University of Münster, D-48149 Münster, Germany; gerrit.frommeyer@ukmuenster.de (G.F.); lars.eckardt@ukmuenster.de (L.E.); 2 Heart Center Ludwigshafen, Department of Cardiology, D-67063 Ludwigshafen, Germany; kleemant@klilu.de; 3 Department of Cardiology, Klinikum Coburg, D-96450 Coburg, Germany; johannes.brachmann@regiomed-kliniken.de; 4 Department of Cardiology, Medical University Hospital, D-69120 Heidelberg, Germany; patrick.lugenbiel@med.uni-heidelberg.de; 5 Department of Cardiology and Internal Intensive Care Medicine, Heart Center Munich-Bogenhausen, D-81925 Munich, Germany; sebastian.reif@muenchen-klinik.de; 6 Stiftung Institut für Herzinfarktforschung (IHF), D-67063 Ludwigshafen, Germany; hochadel@ihf.de (M.H.); senges@stiftung-ihf.de (J.S.)

**Keywords:** cardiac resynchronization therapy, ICD implantation, cardiovascular mortality, German device registry

## Abstract

**Background**: According to current guidelines, cardiac resynchronisation therapy (CRT) is recommended in patients with significantly impaired left ventricular systolic function and left bundle branch block. However, the decision between pacemaker (CRT-P) and defibrillator (CRT-D) in patients eligible for CRT remains a matter of debate. Register data have shown a higher all-cause mortality in CRT-P in comparison to patients with a CRT-D. Here, we investigated clinical determinants of the selection of CRT-P vs. CRT-D and clinical outcome in large registry data from a multi-centre ‘real-life’ registry on patients with CRT defibrillator or pacemaker therapy. **Methods**: The German Device Registry (DEVICE) is a nationwide, prospective registry with one-year follow-up investigating 5451 patients receiving device implantations in 50 German centres. The present analysis of DEVICE focused on all patients from the register receiving cardiac resynchronization therapy. **Results**: Out of 1603 patients receiving cardiac resynchronisation therapy, 1536 (95.8%) received a CRT defibrillator system and 67 (4.2%) a CRT pacemaker system. Patients in the CRT-P group had a significantly better left ventricular systolic function compared to the CRT-D group (median 35% vs. 25%), and significantly less often had a history of myocardial infarction (9.0% vs. 25.2%). A preexisting pacemaker and a history of stroke/peripheral embolism were identified as determinants for the selection of CRT-P vs. CRT-D. Overall mortality after one-year follow-up was 8.0%. Patients with ischemic cardiomyopathy receiving CRT-P therapy had a higher one-year mortality than patients receiving CRT-D (21.2% vs. 8.9%, *p* = 0.020). On the other hand, patients with non-ischemic cardiomyopathy did not display differences between these two treatment groups (CRT-P 8.1%, CRT-D 6.6%, *p* = 0.72). **Conclusions**: Data from the German device registry show that most patients receiving cardiac synchronization therapy have an implanted CRT-D system. In comparison to patients with CRT-D, those with CRT-P more often had a non-ischemic cardiomyopathy and a preexisting pacemaker system. The outcomes between these two treatment groups were different as regards ischemic cardiomyopathy only.

## 1. Introduction

In patients with heart failure and reduced left ventricular ejection fraction, device-based therapies including cardiac resynchronisation therapy (CRT) and implantable cardioverter–defibrillator (ICD) therapy are recommended in the current guidelines [1], and have a favourable impact both on morbidity and on mortality [2,3,4]. However, in patients who qualify for CRT, the choice between a CRT pacemaker (CRT-P) and CRT defibrillator (CRT-D) is still the subject of controversial debate. In the absence of large, prospective randomized clinical trials directly comparing CRT-P and CRT-D, small observational clinical studies [5] and data from registries [6] suggest an increased long term survival of patients receiving CRT-D. In current practice, most CRT patients an implanted defibrillator, which is considered a supposed mortality benefit associated with ICD therapy. However, the role of a defibrillator is not well established in this setting [7,8,9]. When CRT is indicated, patients with large myocardial scars might benefit from concomitant ICD therapy. In contrast, patients with relevant comorbidities and non-ischemic cardiomyopathies are possibly more likely to benefit from a CRT-P system with less risk of lead complications, reduced costs, and lack of inappropriate ICD shocks. In the present study, data from a multi-centre real-world registry on patients receiving CRT were analyzed and characteristics and outcomes of patients receiving CRT pacemaker therapy were compared to patients receiving CRT defibrillator therapy.

## 2. Methods

The German Device Registry has already been described elsewhere [10,11]. Briefly, the German Device Registry is a nationwide, prospective database of cardiac device (ICD or CRT) implantations and revisions organized by the Stiftung Institut für Herzinfarktforschung Ludwigshafen, Germany (IHF). Fifty participating centres documented information on demographic data, device indication, implantation procedure, and peri-interventional complications at the time of device implantation. The recruitment of patients for the German Device Registry started in March 2007 and lasted until March 2011, and then continued as Device II registry, which was terminated in February 2014. After written informed consent had been obtained, data were entered into an internet-based electronic case report form by the centres. Case report forms were thereafter transmitted encrypted with a secure socket layer, and the IHF took responsibility for data management and monitoring.

The present study includes all patients from the register receiving cardiac resynchronisation therapy (both defibrillator and pacemaker). There were no exclusion criteria in the present analysis. At baseline, demographic data, details on cardiac disease, implanted devices, ECG parameters, and comorbidities were noted. Patients received the device according to the decision and standards of the participating centres. Follow-up contacts were scheduled one year after implantation or revision by telephone. The follow-up was performed by the IHF. During telephone contact questions on arrhythmias (e.g., syncope, resuscitation, ablation), cardiac events (e.g., myocardial infarction, revascularization), complications, medication, and heart failure symptoms were posed. In case of an ineffective call, further information was gathered from other caring physicians or civil registration offices.

### Statistical Analysis

The patient population is characterized by descriptive statistical measures. Categorical data are presented as percentages and metrical data as medians with 25th and 75th percentiles. The distribution of binary variables was compared between age groups using a Pearson Chi-square test, and that of metrical variables by a Mann–Whitney test. The baseline data shown are 99% complete, except where indicated. The descriptive statistics are based on the available cases. Observation time was calculated as the time span from the index intervention to the last follow-up contact. One-year all-cause mortality up to 365 days after the index procedure was estimated by the Kaplan–Meier method and cardiovascular mortality by a competing risks approach. Adjusted and unadjusted hazard ratios were calculated by Cox regression. The statistical computations were performed using SAS release 9.4 on a personal computer (SAS Institute, Inc., Cary, NC, USA).

## 3. Results

### 3.1. Patient Characteristics/Demographics

Demographic characteristics are summarized in Table 1. Out of 5451 patients registered, 1603 patients met the inclusion criteria. Of those, 67 (4.2%) received a CRT-P and 1536 patients (95.8%) received a CRT-D system. The median age of the CRT-P group was 74 years, and it was 70 years in the CRT-D group (*p* < 0.001). Overall, 74.6% of the CRT-P patients were male, and 78.6% of the CRT-D patients. Additionally, 75.8% of the CRT-P group were in NYHA class III or higher, while 81.2% of CRT-D patients presented with NYHA class III or higher (no significant difference). Patients in the CRT-P group had a significantly better left ventricular systolic function compared to the CRT-D group (median 35% vs. 25%). Patients in the CRT-P group had a history of myocardial infarction significantly less often (9.0% vs. 25.2%). COPD was more common in CRT-P patients than in CRT-D patients (13.4% vs. 5.0%); however, this difference did not reach the level of statistical significance. A history of peripheral embolic events was significantly more common in patients receiving CRT-P therapy. The indication for defibrillator therapy was primary prevention in 81.1% and secondary prevention in 18.9% of the CRT-D patients. Sinus rhythm was significantly less common in the CRT-P group, and QRS duration was significantly higher in the CRT-P group. Atrial fibrillation (AF) was more common in CRT-P versus CRT-D patients (28.4% vs. 19.2%; *p* = 0.065). ECG findings and further details regarding comorbidities are summarized in Table 1. In a regression analysis (C = 0.835), predictors for the selection of CRT-P vs. CRT-D were age, a preexisting pacemaker, and a history of stroke and peripheral embolism, whereas a low LVEF and prior myocardial infarction predicted CRT-D therapy (Table 2). The presence of atrial fibrillation was not predictive for system selection.

### 3.2. Implantation Procedure and In-Hospital Treatment

In-hospital complications were more common in the CRT-P group (13.7%; information available from 51 patients) than in the CRT-D groups (5.9%, *p* = 0.033). The cardiovascular medication at the time of discharge is displayed in Table 3. Of note, CRT-P patients received ACE inhibitors, beta blockers, and aldosterone antagonists significantly less often compared to CRT-D.

### 3.3. Follow-Up

Documented follow-up information was obtained for 65 (97%) of the 67 patients in the CRT-P group at a median observation time of 17.4 months (13.9; 23.3), and for 1489 patients in the CRT-D group at a median observation time of 17.2 months (13.1; 24.5). Table 4 details the medication at follow-up. In the CRT-P group, 56 patients were alive at their last contact, while 11 patients had died during their observation period (survival time: 6.4 months (1.6; 10.5)). In the CRT-D group, 1298 patients were alive at their last contact, while 238 patients had died (survival time 12.5 months (4.7; 33.1); *p* = 0.059 compared to the CRT-P group) during follow-up. These numbers correspond to a 1-year mortality of 13.8% vs. 7.8% (Kaplan–Meier analysis; *p* = 0.076 (log-rank test)). The combined endpoint of death, myocardial infarction, stroke (MACCE) was observed in 15.4% of the CRT-P and in 8.8% of the CRT-D group (*p* = 0.070).

During follow-up, syncope occurred significantly more often in the CRT-P group (21.9%; data available from 32 patients) than in the CRT-D group (4.3%; data available from 770 patients; *p* < 0.001). In the CRT-D group, 12% received adequate ICD shocks. Four patients (47.2%) died of unknown cause. Additionally, 25 deaths (34.7%) were classified as cardiovascular while 13 deaths (18.1%) were non-cardiovascular deaths. The estimated 1-year overall mortality was 5.4%. The rate of major adverse cardiac and cerebrovascular events (MACCE; including death, myocardial infarction, and stroke) was 6% (n = 45) after one year. A total of 77 patients (10.9%) experienced shock deliveries of the ICD. Syncope occurred in 15 patients (2.1%) and electrical storm was documented in 9 patients (1.6%).

In an additional analysis, patients with ischemic and non-ischemic cardiomyopathy were analyzed separately (Table 5 and Table 6). Patients with ischemic cardiomyopathy (ICM, Table 5) receiving CRT-P therapy had a higher one-year mortality than patients receiving CRT-D (21.2% vs. 8.9%, *p* = 0.020). One-year MACCE (death, myocardial infarction, stroke) was also higher in ICM CRT-P patients in comparison to CRT-D patients with an underlying ICM (25.0% vs. 10.1%, *p* = 0.012). For ICM, the in-hospital complication rate was also higher in CRT-P vs. CRT-D groups (25.0% vs. 6.1%, *p* = 0.004).

On the other hand, patients with non-ischemic cardiomyopathy (NICM) did not display significant differences with regard to one-year mortality, MACCE, or in-hospital complications (Table 6).

**Figure 1:**
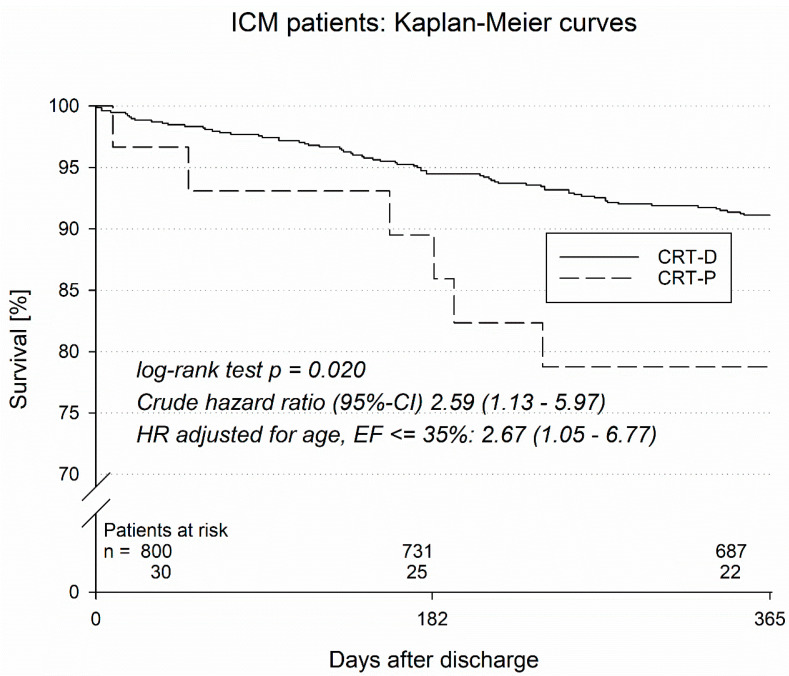
Kaplan–Meier curves of ICM patients.

**Figure 2:**
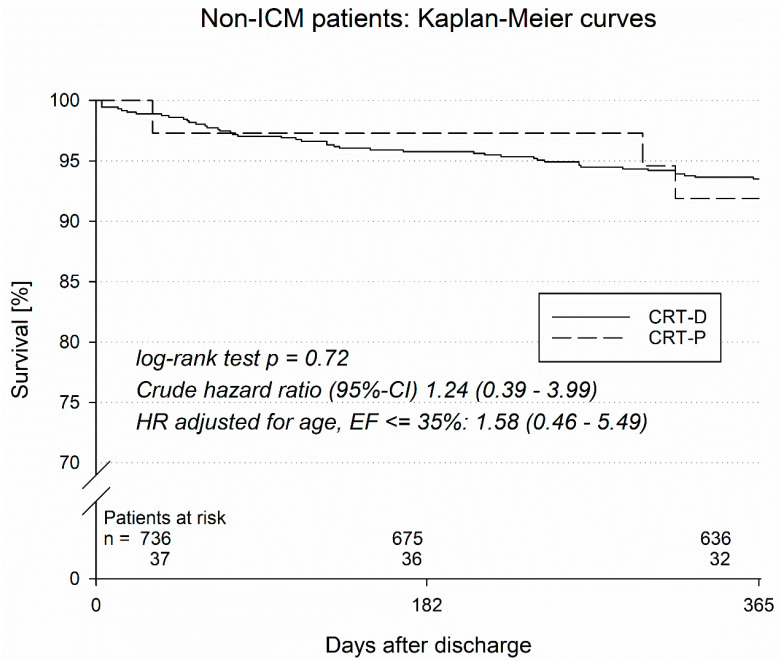
Kaplan–Meier curves of non-ICM patients.

In a Kaplan–Meier analysis, the survival of CRT patients with ischemic and non-ischemic cardiomyopathy was analyzed (Figure 1 and Figure 2). Patients with ischemic cardiomyopathy (ICM) with a CRT-D implanted had significantly better survival (log rank test *p* = 0.020) than ICM patients with an implanted CRT-P system. Specifically, CRT-P vs. CRT-D therapy in ICM patients was associated with a crude hazard ration (95%-CI) of 2.59 (1.13–5.97); when the hazard ration was adjusted for age, LVEF ≤ 35% amounted to 2.67 (1.05–6.77). On the other hand, patients with non-ischemic cardiomyopathy (NICM) with an implanted CRT-D system did not have a better survival rate when compared to NICM CRT-P patients (log rank test *p* = 0.72; crude hazard ratio (95%-CI) 1.24 (0.39–3.99)), with a hazard ratio adjusted for age of LVEF ≤ 35% 1.58 (0.46–5.49).

## 4. Discussion

The present study provides insights into the real-world clinical practice of device choice for patients eligible for cardiac resynchronization therapy (CRT). The present results of the German device registry analysis suggest that most clinicians prefer to implant CRT defibrillators. Only a small minority of patients received a CRT pacemaker.

In a contemporary patient cohort, primary prevention ICD implantation led to a 27% lower mortality, with similar results in patients with ischemic and non-ischemic cardiomyopathy (EU-CERT-ICD trial) [12]. However, in this trial, only patients with narrow QRS were included. On the other hand, CRT itself may confer antiarrhythmic effects. In a recent meta-analysis [13], Saini et al. found that CRT responders had a significant reduction in ventricular arrhythmias compared with non-responders. Of note, CRT nonresponse was associated with a significant increase in ventricular arrhythmia risk. Several small clinical studies and data from the multicentre registry suggest that long-term survival was better in CRT-D patients than in CRT-P patients. Like in our study, CRT-D and CRT-P patients presented with marked differences in baseline characteristics, but multivariate analyses showed a substantially lower mortality in these studies.

In the Comparison of Medical Therapy, Pacing, and Defibrillation in Heart Failure Trial [14], only CRT-D was associated with a significantly lower overall mortality in comparison to controls. The post hoc analysis directly comparing CRT defibrillator and pacemaker therapy demonstrated a more effective reduction in sudden cardiac death risk in CRT defibrillator patients compared to CRT pacemaker patients. However, this difference did not translate into a substantial and significant overall survival benefit.

Current ESC guidelines [1] do not provide clear advice on whether patients with an indication for CRT should receive a pacemaker or defibrillator implantation, but advocate for careful evaluation of the potential benefits of CRT in patients with ICD indication before implantation. The guidelines state that the role of the additional defibrillator is less well established. Therefore, many physicians decide based on individual patient advice, taking into consideration the likelihood of ventricular arrhythmias, age, and comorbidities, and hence individual life expectance. In our registry, only age, a preexisting pacemaker, and a history of stroke and embolism were predictors for CRT-P therapy. A smaller clinical study based on a prospective multicentre Italian registry [5] found that patients receiving CRT-P were predominantly older, female, had no history of ventricular arrhythmias, and more frequently had a non-ischemic etiology of heart failure. In our registry, the only predictor of death from any cause was CRT pacemaker therapy. A higher rate of comorbidities such as peripheral embolic events is one possible explanation. Moreover, age, a pre-existing pacemaker, and stroke/peripheral embolism were identified as determinants for the selection of CRT-P vs. CRT-D and might confer a higher risk for all-cause mortality.

In a single-centre study by Martens et al. [6], a higher all-cause mortality was reported in patients with CRT-P in comparison with CRT-D after adjusting for baseline characteristics. Interestingly, the mode of death analysis revealed a predominant non-cardiac mode of death in CRT pacemaker recipients.

Thus, combining cardiac resynchronization therapy with ICD therapy could shift the mode of death from arrhythmia-caused mortality to cardiac pump failure, and possibly even to non-cardiac modes of death. In the register data presented here, CRT-P recipients were significantly older than CRT-D recipients; therefore, conclusions with regard to mortality cannot be drawn reliably.

When comparing the survival of CRT-D and CRT-P recipients separately with regard to the type of cardiomyopathy, a relevant difference was observed in ischemic cardiomyopathy only. The benefit of CRT-D therapy vs. CRT-P therapy in patients with ICM continued to be present even after adjustments for age and LVEF ≤ 35%. However, due to the small number of CRT-P patients, these results must be interpreted with caution.

In general, it is assumed that the potential benefits of ICD therapy for primary prevention depend on the type of cardiomyopathy underlying the impairment of systolic left ventricular dysfunction. In a recent meta-analysis of combined data from five landmark ICD trials, ICM patients experienced a similar risk of ventricular arrhythmia in comparison to NICM patients, but demonstrated an increased risk of all-cause mortality [15]. However, the NICM patients in this meta-analysis had a significantly greater QRS duration than ICM patients. Since NICM patients were more likely to have a CRT-D present at enrollment, the authors studied the association between CRT-D and all-cause mortality and found that the association between ICM and all-cause mortality persisted for patients with and without CRT-D at enrollment.

Interestingly, the results of the recent DANISH trial [16] suggest that ICD implantation in patients with non-ischemic heart disease do not result in an overall benefit in terms of mortality. As one possible explanation, it has been argued that a high proportion of patients in the DANISH trial had received CRT, suggesting that CRT alone confers a substantial survival benefit in these patients. Moreover, subanalyses based on patient age revealed that patients aged 68 years or older did not benefit, while in younger patients, below 59 years, beneficial effects of ICD therapy were observed [16]. Of note, according to a recent survey of the European Heart Rhythm Association, results of the DANISH study have already changed clinical decisions in Europe [17].

## 5. Limitations

Due to the nature of a registry database, the present study has several limitations. In addition to the potential selection bias of the registry, the data quality is not comparable to the quality of an international randomized controlled study. The number of CRT-D patients greatly outnumbers the patient number in the CRT-P group, and both patient groups differ significantly in several key characteristics. The differences in age, comorbidities, and left ventricular ejection fractions do not allow a direct comparison of the outcome. In addition, there is no other control group in this registry. Of note, the follow-up duration of about one year does not provide information on the long-term outcome of both groups. In fact, the limited number of CRT-P patients in combination with the short follow-up may impair the power of the study in such a way that significant differences in the clinical outcome of both groups are not detected. Moreover, while increased mortality and MACCE events were observed in CRT-P patients with underlying ischemic cardiomyopathy compared to those who received CRT-D with the same etiology, the former patient group was also on significantly lower goal-directed medical therapy than the latter. In addition, patients receiving CRT-D were younger than those receiving CRT-P. Therefore, a confounding effect with regard to the follow-up analysis cannot be ruled out. Also, a bias in the way the devices are funded is possible.

Nonetheless, the results of the present study represent ‘real-life’ data in patients receiving cardiac resynchronization therapy. The data partially confirm previous data from registries and small clinical trials demonstrating an elevated mortality and morbidity rate in patients receiving CRT pacemaker therapy, even if the difference did not reach the level of statistical significance.

## 6. Conclusions

The present data from the German Device registry demonstrate that in patients eligible for CRT, pacemakers are preferentially implanted in elderly patients with more comorbidities and non-ischemic forms of cardiomyopathy. In contrast, patients receiving CRT-D were younger, more often had a history of myocardial infarction, and had a poorer LV ejection fraction. These registry data thereby reflect clinical decision making in a real-life scenario. The data imply that the decision for CRT pacemaker therapy was made only in a minority of cases. In the short-term follow-up, CRT pacemaker patients with non-ischemic cardiomyopathy did not display a higher mortality compared to non-ischemic cardiomyopathy patients receiving a CRT defibrillator. Therefore, it seems reasonable to administer CRT pacemaker implantation in selected, elderly patients, preferably with non-ischemic forms of cardiomyopathy and more comorbidities, without having to accept an excessively higher mortality. 

## Figures and Tables

**Table 1:** Patient characteristics.

	CRT-P (n = 67)	CRT-D (n = 1536)	*p* Value
Age (years)	74 (70; 80)	70 (62; 75)	<0.001
Male	74.6%	78.6%	0.44
CHD	44.8%	52.1%	0.24
MI	9.0%	25.5%	0.002
DCM	47.8%	52.0%	0.49
HCM	1.5%	0.8%	0.85
Diabetes	35.8%	31.3%	0.43
Hypertension	59.7%	56.0%	0.55
COPD	13.4%	5.0%	0.003
Renal insufficiency	25.4%	24.2%	0.82
Stroke	9.0%	4.1%	0.056
Peripheral arterial disease	4.5%	3.2%	0.56
Peripheral embolic event	6.0%	1.7%	0.011
Heart rate (bpm)	68 (62; 80)	70 (60; 80)	0.59
Blood pressure (mm Hg sys.)	124 (110; 140)	120 (113; 135)	0.63
Blood pressure (mm Hg dias.)	70 (70; 80)	76 (70; 80)	0.84
QRS duration (ms)	162 (140; 180)	160 (140; 180)	0.18
QRS duration > 150 ms	68.7%	56.1%	0.042
LBBB	76.1%	82.8%	0.16
RBBB	3.0%	3.2%	0.92
NYHA class in underlying cardiac disease			
NYHA I	6.5%	2.1%	
NYHA II	17.7%	16.8%	
NYHA III	69.4%	74.4%	
NYHA IV	6.5%	6.7%	
NYHA III+	75.8%	81.2%	0.29
LVEF	35 (27; 40)	25 (20; 30)	<0.001
LVEF ≤ 30%	42.6%	79.3%	<0.001
Sinus rhythm	62.7%	74.7%	0.028
Atrial fibrillation	28.4%	19.2%	0.065

CHD: Coronary heart disease, MI: myocardial infarction, DCM: dilatative cardiomyopathy, HCM: hypertrophic cardiomyopathy, COPD: chronic obstructive pulmonary disease, LBBB: left bundle brunch block, RBBB: right bundle brunch block, LVEF: left ventricular ejection fraction.

**Table 2:** Determinants for the selection of CRT-P vs. CRT-D.

	Adjusted OR (95%-CI)	*p* Value
Age [each 10 years]	2.82 (1.57–3.31)	<0.001
Female sex	1.04 (0.55–1.99)	0.90
Preexisting pacemaker	3.13 (1.58–6.23)	0.001
Stroke/peripheral embolism	2.42 (1.05–5.58)	0.039
COPD	2.21 (0.89–5.47)	0.086
EF ≤ 35%	0.12 (0.06–0.22)	<0.001
Myocardial infarction	0.19 (0.07–0.53)	0.002
Atrial fibrillation	1.04 (0.55–1.95)	0.91

C = 0.835.

**Table 3:** Medication at discharge from hospital.

	CRT-P (n = 67)	CRT-D (n = 1536)	*p* Value
ACE-I/AT1-antagonists	82.1%	91.9%	0.005
Betablocker	82.1%	91.6%	0.007
Aldosterone antagonists	22.4%	49.0%	<0.001
Diuretics	80.6%	84.5%	0.38
Class I AAT	0%	0.6%	0.53
Class III AAT	16.4%	14.4%	0.65
Class IVAAT	6.0%	4.2%	0.48
Digitalis	20.9%	25.5%	0.40

AAT: Antiarrhythmic therapy.

**Table 4:** Medication at follow-up.

	CRT-P (n = 45)	CRT-D (n = 1044)	*p* Value
ACE-I/AT1-antagonists	80.0%	85.9%	0.27
Betablocker	80.0%	90.1%	0.028
Aldosterone antagonists	20.0%	52.3%	<0.001
Diuretics	71.1%	78.9%	0.21
Class I AAT	0%	0.7%	0.58
Class III AAT	15.6%	17.1%	0.79
Class IVAAT	4.4%	6.0%	0.66
Digitalis	8.9%	25.0%	0.014

AAT: Antiarrhythmic therapy.

**Table 5:** Complication rate and outcome of patients with ischemic cardiomyopathy (ICM).

ICM	CRT-P (n = 30)	CRT-D (n = 800)	*p* Value
In-hospital complications requiring intervention	6.7% (2/30)	2.8% (22/795)	0.22 *
One-year mortality rate ^†^	21.2%	8.9%	0.020
One-year MACCE (death, stroke, myocardial infarction)	25.0% (7/28)	10.1% (79/781)	0.012

* Fisher’s exact test. ^†^ Rate calculated by Kaplan–Meier method, *p*-value by log-rank test.

**Table 6:** Complication rate and outcome of patients with non-ischemic cardiomyopathy (NICM).

NICM	CRT-P (n = 37)	CRT-D (n = 736)	*p* Value
In-hospital complications requiring intervention	5.4% (2/37)	2.6% (19/732)	0.27 *
One-year mortality rate ^†^	8.1%	6.5%	0.72
One-year MACCE (death, stroke, myocardial infarction)	8.1% (3/37)	7.3% (52/708)	0.86

* Fisher’s exact test. ^†^ Rate calculated by Kaplan–Meier method, *p*-value by log-rank test.

## Data Availability

Research Data cannot be shared due to ethical/privacy restrictions.
